# Effectiveness and Safety of Laser-Assisted Removal of Inferior Vena Cava (IVC) Filters in a Single Tertiary Care Center

**DOI:** 10.7759/cureus.32809

**Published:** 2022-12-21

**Authors:** Abdulmohsen Alhussaini, Mohammed A Alahmad, Mohammad M Alomaim, Mohammed Y Alzahrani, Abdullah S Alghamdi, Mohammad Arabi

**Affiliations:** 1 Radiology, King Abdulaziz Medical City Riyadh, Riyadh, SAU; 2 Medical Intern, King Saud Bin Abdulaziz University for Health Sciences College of Medicine, Riyadh, SAU; 3 Interventional Radiology, King Abdulaziz Medical City Riyadh, Ministry of National Guard Health Affairs, Riyadh, SAU

**Keywords:** ivc filter retrieval, interventional radiology, inferior vena cava filter, filter removal, embedded, complex filter removal, ivc filter

## Abstract

Introduction: Laser sheath-assisted removal of inferior vena cava (IVC) filters with long dwelling time is a technique that utilizes laser-tipped sheaths. The laser light only penetrates vascular tissue by one hundred microns, causing the target tissues to disintegrate into particles less than 5 microns in size. This approach reduces the energy used during difficult retrieval procedures, allowing permanent filters to be removed in less fluoroscopic and procedural time overall.

Materials and methods: The radiology information system and electronic health records were used in this retrospective cohort study to retrieve the data. A total of nine consecutive patients who underwent laser-assisted filter removal utilizing GlideLightTM were included in the study between January 2016 and January 2017. The study took place at King Abdulaziz Medical City in Riyadh. In this study, five patients were male and four were female with ages ranging from 19 to 57 years with a median age of 31.

Results: During the period of the study, a total of nine patients had their IVC filters removed using a laser. The success rate was 100%. The indications were trauma (n=4) followed by deep vein thrombosis (DVT) (n=3) and one patient indication was prolonged immobilization. The dwelling time ranged from seven to 70 months, with a dwelling median of 19 months.

Conclusion: A laser sheath might be necessary for closed-cell filters in order to improve the likelihood of a successful and secure retrieval. Technical efficiency, filter type, the necessity of applying a laser sheath based on an open versus closed filter design, dwell times, and unfavorable results. As a result, after typical procedures failed to successfully retrieve IVC filters with long dwell durations, laser-assisted filter removal is thought to be practical and safe.

## Introduction

Inferior vena cava (IVC) filters have been proven effective in preventing primary and recurrent pulmonary embolism for high-risk patients of venous thromboembolic diseases [1]. Initially, it was placed with open surgery; however, due to advancements in medicine, it is performed endovascularly by utilizing fluoroscopy [2]. Thromboembolic events remain a significant reason for mortality and morbidity [3]. When anticoagulation is contraindicated, placing a filter in the IVC in the case of deep vein thrombosis (DVT) is crucial for preventing pulmonary embolism (PE) [3]. IVC filters do, however, carry an increased risk of consequences, including both thrombotic and non-thrombotic events, with prolonged usage [4]. When the filter is no longer clinically necessary, it should be removed. Otherwise, filters risk becoming deeply lodged in the endothelium, making retrieval much more challenging. As the filter dwell time gets longer, certain well-known problems related to unrecovered IVC filters occur more frequently. Some of these side effects include caval wall penetration, filter breakage or migration, caval thrombosis, and an increased risk of lower extremity DVT [5]. The current increase in difficulties associated with filters results from low retrieval rates for IVC filters (35-38.36% retrieval rate) [6]. Departmental registries resulted in an annual growth rate of 11.6-18.1% in retrieval rates [6]. Standard retrieval techniques cannot be used to remove filters all the time since they may put many patients at risk for complications associated with the filters themselves [7].

Advanced methods are typically necessary to maintain appropriate removal rates. When employing the conventional snare technique, filter recovery was demonstrated to be effective in about 80-90% of situations [8]. However, additional retrieval techniques are required when a snare cannot catch the filter hook [8]. The use of supplementary and innovative removal methods rises when the filter retrieval demonstrates filter relocation, fracture, extra endothelial growth, or thrombus [8,9]. A single-institutional prospective analysis found a 98% success rate using a laser to remove the IVC filter from the surrounding endothelial and fibrotic tissue [10]. This technique utilizes controlled photothermal ablation of the endothelium surrounding the filter struts using a GlideLightTM laser-tipped sheath (Spectranetics, Colorado Springs, CO, USA) powered by a 308 nm xenon chloride excimer laser generator (CVX-300; Spectranetics). The laser light only penetrates 100 microns into vascular tissue triggering the target tissues to break into bits smaller than 5 microns. This method decreases the total energy used through complex recovery procedures, letting permanent filters detach in the less fluoroscopic and procedural period. 

## Materials and methods

This retrospective study received approval from King Abdullah International Medical Research Center, Riyadh, Saudi Arabia (IRB/0057/22) and informed consent was not required. The radiology information system and electronic health records were used to retrieve the data. The study comprised nine consecutive patients who had their IVC filters removed using a laser after routine retrieval efforts failed or when the filters were otherwise deemed non-retrievable due to anticipated procedure difficulties. Patients ranging in age from 19 to 57 years were included in the study, which comprised five males and four females (with a median age of 31 years). The dwelling time ranged from seven to 70 months, with a dwelling median of 19 months (Table 1). The most prevalent indication was trauma (n=4) followed by DVT (n=3) and one patient indication was acute leukemia post bone marrow transplant and one after internal fixation of the knee. Eight patients had Optease filters (Cordis, Miami Lakes, FL, USA), and one had a Recovery filter (Bard, Murray Hill, NJ, USA). Pre-intervention findings were positive for two of the patients. One patient had typical iliac vein occlusion, and one had IVC occlusion secondary to an IVC filter. 

**Table 1:** Demographics of the patients, technique used DVT: deep vein thrombosis; IVC: inferior vena cava

Pt Number	Gender	Age	Indication	Dwelling time (in months)	Filter Type	Access	Pre-OP findings
1	Male	21	Trauma/prophylactic	7	Optease	Femoral	None
2	Male	23	Trauma/prophylactic	24	Optease	Femoral	None
3	Male	19	Trauma/prophylactic	20	Optease	Jugular	Common iliac occlusion
4	Female	20	Pregnancy/DVT	19	Optease	Femoral	None
5	Female	46	DVT/Bleeding	70	Recovery	Jugular	None
6	Female	35	DVT/Bleeding	12	Optease	Femoral	None
7	Female	53	Acute leukemia post bone marrow transplant	10	Optease	Femoral	None
8	Male	31	Trauma/prophylactic	39	Optease	Three accesses (right internal jugular and bilateral femoral)	IVC occlusion
9	Male	57	After internal fixation of the knee	12	Optease	Jugular and Femoral	None

The primary study objectives were defined as follows: complete filter detachment from the vena cava wall and removal from the body, excluding extravascular filter fragments, was considered technically successful. Technical failure was defined as the presence of major procedure‐related complications, and the difference in force applied to a patient's filter both with and without laser assistance during attempted filter removal. Secondary objectives were defined as the resolution of symptoms in patients with filter‐related morbidity. The primary outcome was successful filter retrieval.

All procedures were done under general anesthesia. Filter removal was performed via femoral vein access (n=5) and jugular vein (n=2); two patients required both accesses. Removal procedures require various techniques, including retrieval cones, snares, wire loops, and snares. All procedures were done using a tri-axial system using an outer 14-18 Fr sheath to allow the insertion of the laser-tipped sheath (12-16 Fr) that allows the introduction of the retrieval tools. The laser sheath has a 50 cm working length and is calibrated to deliver 60mJ/mm2 before insertion. Once the filter is correctly captured into the sheath tip, the laser sheath is activated intermittently to ablate the tissue immediately facing the tip of the sheath. Sheath advancement should be slow to allow efficient tissue ablation. To decrease the risk of problems, the filter is separated from the IVC wall using a combination of delicate dissection utilizing the laser and alternating tissue ablation. Patients were prescribed therapeutic doses of low molecular heparin for 10 days while bridging to oral anticoagulation for three to six months. Patients were clinically followed up at one month and with cross-sectional imaging to assess for potential complications.

## Results

**Figure 1:**
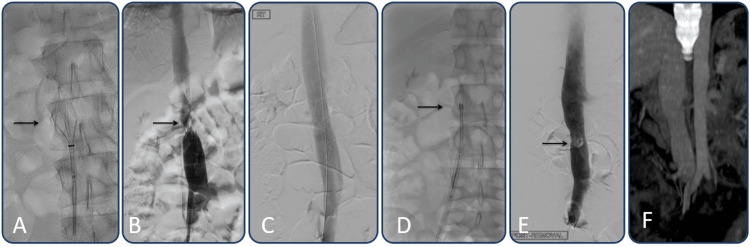
A 23-year-old trauma patient who had an inferior vena cava (IVC) filter placed for prophylaxis prior to orthopedic surgery A) Radiograph shows a partially collapsed Optease filter (arrow) during the initial retrieval attempt. The filter could not be retrieved despite excessive force application. B) Post unsuccessful removal attempt venography showed a high-grade stenosis (arrow) due to IVC collapse during the retrieval procedure. C) Post-balloon angioplasty venography shows patent IVC and resolution of the stenosis. D) On a different session, a 12 Fr GlideLightTM laser sheath (arrow) was introduced, through a 14 Fr outer sheath, and intermittently activated to gradually collapse the filter. E) Post removal venography shows a residual filling defect (arrow) that may represent a fibrin sheath or acute thrombus. F) Coronal reconstruction of a CT scan of the abdomen two months later shows complete resolution of the filling defect with no residual stenosis.

All in all, filter removal assisted by a laser sheath was technically successful (100%). Following retrieval, five patients developed minimal IVC stenosis, which required balloon angioplasty in four (Figure 1).

One patient had slight extravasation and did not require treatment. Follow-up imaging showed recurrent IVC occlusion in the patient who presented initially with IVC occlusion. The patient who came with a typical iliac occlusion had little filter fragments implanted in the wall at the initial site. The remaining patients had no IVC abnormality on follow-up imaging and showed patent IVC (77%). The average cumulative fluoroscopic time which is a standard metric used for clinical radiation management was 55 minutes ranging from 3 to 190 minutes. The average of the total dose area product (DAP) which is defined as the dose absorbed and the area irradiated was 279196 mGycm2 ranging from 5646 mGycm2 to 1765730 mGycm2 (Table 2). Patients were prescribed prophylactic enoxaparin for 10 days post-procedure. All patients discontinued the anticoagulation therapy.

**Table 2:** Outcomes of the patients IVC: inferior vena cava; DAP: dose area product *Patient didn't show up in the follow-up

Pt Number	Outcome	Average cumulative fluoroscopic exposure time (Minutes)	Total DAP (mGyCm^2^)	Complications	Post removal findings	Follow up CT
1	Successful	3	5646	None	Small luminal filling defect and mild narrowing	Patent IVC
2	Successful	44	73255	None	Patent	Patent IVC
3	Successful	117	225329	None	IVC stenosis	Patent IVC tiny filter spikes remained embedded in the wall, recurrent right CIV occlusion with no symptoms
4	Successful	57	83741	None	Minimal focal stenosis	Patent IVC
5	Successful	14	32861	None	Patent	Patent IVC
6	Successful	13	112647	Limited extravasation	Limited extravasation and focal narrowing	Patent IVC
7	Successful	48	169865	None	Minimal focal stenosis	Patent IVC
8	Successful	190	1765730	None	Remaining fragments within the wall	Fragmented residual IVC below the level of the renal veins with complete occlusion of the infra renal IVC with multiple collaterals
9	Successful	11	43692	None	Chronic fibrotic fibrin in the wall	No follow up*

## Discussion

Pharmacological anticoagulation is the first line of management in case of venous thromboembolic events. Still, in some cases, there are contraindications for the use of anticoagulation. IVC filters are used very frequently - up to 25 times more often than a comparable population in Northern Europe [10]. According to a recent analysis of the US National Inpatient Sample, over 1.1 million devices were implanted over a 10-year period [11]. This high implantation rate, combined with historically low retrieval rates and sparse clinical follow-up [12-14], has contributed to the current rise in filter-related problems [11,15]. The use of advanced techniques was associated with a higher success rate of 95% versus 73% but also a significantly higher complication rate of 5.3% versus 0.4%, according to a 2014 study comparing 231 routine retrieval attempts with 57 advanced retrieval attempts (mean dwell time=277 days for advanced attempts) [16]. Another study with a sample size of 500 patients found that the laser-assisted removal of inferior vena cava filter technique had a 99.4% success rate. Less than 2% of instances suffered major issues [17]. Our study found no significant complications related to laser sheath-assisted filter removal. Minor complications such as IVC stenosis could be managed with angioplasty. When a filter has been in place for two years, the risk of DVT has doubled due to the increased dwelling time [18].

Due to the rising popularity of retrievable filters and the concurrently rising prevalence of complications, the FDA issued a safety alert in 2010. A 2013 FDA research assessing the risk vs. benefit profile of IVC filter implantation and removal recommended IVC filter recovery within 29 to 54 days following the insertion, once the possibility of PE tapers [19]. Future quality improvement protocols should concentrate on this rising complication rate with retrievable filters, which is probably caused by inadequate follow-up for removal. The idea that removing a filter older than six months is "too hazardous" is not an acceptable excuse to give up.

Our study is limited by its small size and retrospective monocentric nature. Due to the need for more data in Saudi Arabia and Middle East countries, this study will offer a valuable addition to the literature by expressing the experience of a tertiary hospital. Finding the variables that indicate the need for the “laser sheath” for an effective recovery might enhance the patient’s outcomes through assisting in the procedure plan, preventing the need for several retrieval efforts, associated medical expenses, and potential adverse events, as well as increasing the percentage of successful retrievals.

Numerous studies have linked inferior vena cava filters to clinically significant negative consequences. Due to its extensive utilization and propensity for "out of sight, out of mind," standard IVC placement and recovery methods in medical settings must be replaced with a more focused approach. Additional accurate insertion signs, optimal filter selection based on the indication, patient clinical status, acceptable filter technical features, and extra measures promoting proper filter retrieval are all urgently required. Also, the use of this approach outside of tertiary medical facilities may be constrained by the cost of the equipment needed. To maximize the filter's beneficial effects and prevent any long-term issues, understanding the requirement of retrieving filters as quickly as it is practical is very crucial. Establishing a countrywide referral network of institutions for IVC filters whose retrieval has been unsuccessful at the local medical centers is another practical option [20]. Additionally, the Society of Interventional Radiology and the Society of Vascular Surgery is currently conducting the PRESERVE (Predicting Safety and Effectiveness of the Inferior Vena Cava Filters) study, which will result in a prospective, multicenter clinical trial addressing the security and efficiency of IVC filters.

## Conclusions

Complicated factors like long dwelling period of IVC filters can raise technical challenges during the retrieval of IVC filters. Laser-assisted filter removal of IVC filters with long dwelling time showed comparable results with the standard techniques. We report that laser-assisted filter removal is feasible and safe after failed retrieval with standard techniques or in otherwise considered non-retrievable filters with long dwelling time. 
